# [^18^F]LW223 has low non-displaceable binding in murine brain, enabling high sensitivity TSPO PET imaging

**DOI:** 10.1177/0271678X231205661

**Published:** 2023-10-05

**Authors:** Agne Knyzeliene, Mark G MacAskill, Carlos J Alcaide-Corral, Timaeus EF Morgan, Martyn C Henry, Christophe Lucatelli, Sally L Pimlott, Andrew Sutherland, Adriana AS Tavares

**Affiliations:** 1 BHF-University of Edinburgh Centre for Cardiovascular Science, University of Edinburgh, Edinburgh, UK; 2 Edinburgh Imaging, University of Edinburgh, Edinburgh, UK; 3 School of Chemistry, University of Glasgow, Glasgow, UK; 4 West of Scotland PET Centre, Greater Glasgow and Clyde NHS Trust, Glasgow, UK

**Keywords:** [^18^F]LW223, PET, TSPO, *VND*, brain

## Abstract

Neuroinflammation is associated with a number of brain diseases, making it a common feature of cerebral pathology. Among the best-known biomarkers for neuroinflammation in Positron Emission Tomography (PET) research is the 18 kDa translocator protein (TSPO). This study aims to investigate the binding kinetics of a novel TSPO PET radiotracer, [^18^F]LW223, in mice and specifically assess its volume of non-displaceable binding (*V_ND_*) in brain as well as investigate the use of simplified analysis approaches for quantification of [^18^F]LW223 PET data. Adult male mice were injected with [^18^F]LW223 and varying concentrations of LW223 (0.003–0.55 mg/kg) to estimate *V_ND_* of [^18^F]LW223. Dynamic PET imaging with arterial input function studies and radiometabolite studies were conducted. Simplified quantification methods, standard uptake values (*SUV*) and apparent volume of distribution (*V_Tapp_*), were investigated. [^18^F]LW223 had low *V_ND_* in the brain (<10% of total binding) and low radiometabolism (∼15–20%). The 2-tissue compartment model provided the best fit for [^18^F]LW223 PET data, although its correlation with *SUV_90–120min_* or *V_Tapp_* allowed for [^18^F]LW223 brain PET data quantification in healthy animals while using simpler experimental and analytical approaches. [^18^F]LW223 has the required properties to become a successful TSPO PET radiotracer.

## Introduction

The 18 kDa translocator protein (TSPO), is a multifunctional protein expressed in the outer mitochondrial membrane, which plays a role in steroidogenesis, energy metabolism, cell proliferation, apoptosis and immunomodulation.^1,2^ TSPO is also a biomarker of neuroinflammation, as its expression in the brain is upregulated under inflammatory conditions.^3^ Positron emission tomography (PET) imaging of TSPO has been used for non-invasive assessment of neuroinflammation in a number of neurological disorders, such as stroke,^4^ epilepsy,^5^ Huntington’s disease,^6^ Parkinson’s disease,^7^ multiple sclerosis,^8^ and Alzheimer’s disease.^9^ Changes in TSPO expression have also been shown to correlate with disease severity,^10,11^ supporting its diagnostic value.

Unfortunately, the benefits of TSPO PET imaging in neuroscience research and neurology are often obscured by complex data analysis owing to several factors: 1) the rs6971 polymorphism in human *TSPO*,^12^ 2) the need for invasive arterial blood collection to quantify imaging data given the widespread expression of TSPO in organs and blood,^13^ and 3) the poor physicochemical properties of TSPO PET ligands, including high volume of non-displaceable binding (*V_ND_*) in brain tissue^14–16^ and low plasma free fraction (*f_p_*).^17^

Recently, our group reported the discovery of a novel TSPO PET ligand, [^18^F]LW223, which has high affinity for human TSPO (comparable or higher than previously developed compounds, namely PK11195, PBR28 and AB5186), was able to overcome the issues of rs6971 polymorphism sensitivity and has high *f_p_*.^18^ This study aimed to measure [^18^F]LW223 brain *V_ND_* and investigate simplified protocols for quantification of [^18^F]LW223 PET data, in order to rapidly accelerate adoption of the new radiotracer. This is particularly important because the total volume of distribution (*V_T_*), is highly dependent on *V_ND_*. Therefore, high *V_ND_* of TPSO PET radiotracers can significantly impact quantification of TSPO changes in different brain diseases limiting their sensitivity.^19^ Outcome measures derived using a TSPO PET radiotracer with low *V_ND_* and insensitivity to the rs6971 polymorphism would potentially be transformative to the field by enabling accurate quantification of TSPO with greater sensitivity, thus allowing early detection of neuroinflammation and more subtle physiological changes in TPSO expression, such as during natural aging or as a result of subject’s biological sex. This would allow for better understanding of TSPO role both in health and disease. A low *V_ND_* coupled with a simplified, yet accurate, method of quantification of brain TSPO PET could further augment translational potential and widespread use of [^18^F]LW223. We hypothesise that [^18^F]LW223 has low *V_ND_* in murine brain, which will enable high sensitivity TSPO PET imaging.

## Material and methods

### Radiosynthesis of [^18^F]LW223

The radiosynthesis of [^18^F]LW223 was performed as described previously,^18^ except for the mobile phase flow rate used for purification of the final product using a semi-preparative high performance liquid chromatography (HPLC) system, which was reduced from 5 mL/min to 3 mL/min.

### Animals and surgical procedures

All animal experiments were authorised by the local University of Edinburgh Animal Welfare and Ethical Review Committee and in accordance with the Home Office Animals (Scientific Procedures) Act 1986. The ARRIVE guidelines have been used for conducting and reporting animal experiments. The animals were housed under standard 12 h light:12 h dark conditions with food and water available *ad libitum*. Naïve C57Bl/6 adult male mice were used for radiometabolite experiments (n = 31, 19.68 ± 0.90 weeks, 30.18 ± 2.72 g, mean ± SD) and imaging experiments (n = 16, 15.09 ± 4.46 weeks, 28.66 ± 3.38 g, mean ± SD).

For imaging experiments, an intravenous line was established in the femoral vein or tail vein for injection of the radiotracer, and the femoral artery was cannulated to allow automated blood sample collection, as previously described.^20^ For radiometabolite experiments, the femoral artery was cannulated for blood sampling and the radiotracer was administered via the tail vein. Surgical cannulation of the femoral vein and artery was performed as detailed in the supplemental materials.

### Radiometabolite studies

Arterial blood samples and heart, lung, brain, spleen, liver, kidney and adrenal tissue samples were collected at 2, 5, 10, 20, 30, 60 and 120 minutes post-[^18^F]LW223 injection (21.85 ± 8.8 MBq, mean ± SD, n = 31), placed on ice and processed immediately. Blood and tissue samples were processed and analysed as described in the supplemental materials.

### Mass effect study design

To estimate [^18^F]LW223 *V_ND_*, invasive input function data were collected for each mouse (n = 9). An additional cohort of mice (n = 7) scanned dynamically following intravenous bolus administration of [^18^F]LW223 was repurposed from the study by MacAskill et al.^18^ for analysis using simplified outcome measures. Healthy C57Bl/6 adult male mice were anaesthetised using 2% isofluorane gas (Isoflo® APIECE, Zoetis, UK) in a 1 L/min 50/50 oxygen/nitrous oxide mixture. Prior to PET imaging, computed tomography (CT) images were acquired over 5 minutes (nanoPET/CT, Mediso, Hungary). The varying amounts of non-radioactive LW223 in 100% DMSO were injected intravenously together with [^18^F]LW223, as follows: 0.0009 ± 0.0004 mg/kg (n = 3, ‘baseline’), 0.003 ± 0.0008 mg/kg (*n = *6, ‘*Dose 1*’), 0.016 mg/kg (*n = *1, ‘*Dose 2*’), 0.22 ± 0.04 mg/kg (*n = *3, ‘*Dose 3*’), or 0.55 ± 0.09 mg/kg (n = 3, ‘*Dose 4*’) via the catheter established in the femoral vein (*n = *9, current study; 8.6 ± 2.9 MBq, mean ± SD, bolus i.v.) or tail vein (*n = *7 (MacAskill et al. study); 7.3 ± 3.2 MBq, mean ± SD, bolus i.v.). LW223 doses (‘*Dose 1*’ to ‘*Dose 4*’) reported were calculated based on injected volume of pre-made concentrated solution and radiotracer molar activity to provide accurate readouts for the mass effect studies. Following radiotracer injection, mice underwent dynamic PET imaging over 2 hours. At the end of the imaging session, mice received an overdose of isoflurane gas and death was confirmed by cervical dislocation. Whole blood samples (*V_ND_* study only, n = 9) were collected for quantification of [^18^F]LW223 concentration in whole blood and plasma.

### PET/CT acquisition and reconstruction

CT imaging was conducted using semi-circular full trajectories, maximum field of view, 480 projections, 50 kVp, 300 ms exposure time and 1:4 binning. The CT reconstruction was performed using filtered back-projection and the following parameters: 250 × 250 ×250 µm voxel size, cosine filter, 100% cut-off, and corrections for offset, gain and pixel. Dynamic PET imaging was conducted using the 1:5 scanning mode and packet timestamp list mode with a 50% field of view overlap. All PET studies were reconstructed as 0–2 h post-injection imaging datasets. PET studies were reconstructed using the Mediso iterative Tera-Tomo 3-dimensional reconstruction algorithm, which includes point-spread correction, and the following settings: 4 iterations, 6 subsets, full detector model, normal regularisation, spike filter on, voxel size of 0.2 mm, and 400–600 keV energy window. Dynamic PET framing was performed as follows: 1 × 5 s; 17 × 10 s; 1 × 30 s; 1 × 60 s; 2 × 120 s; 10 × 300 s; and 6 × 600 s. All PET data were corrected for randoms, scatter and attenuation.

### Image processing and analysis

Reconstructed PET/CT images were analysed using PMOD version 3.7 (PMOD Technologies, Switzerland). Volumes of interest (VOIs) were manually drawn around the brain, heart, lungs and urinary bladder using CT images. The VOIs of kidneys, adrenals, spleen, liver, gall bladder and intestines were drawn using averaged PET images (0–120 min post-injection). For image-derived input function (IDIF) analysis, spherical 0.8 mm radius VOI was placed in the left ventricle of the heart using averaged PET images, and spherical 1 mm radius VOI was placed in the vena cava using the first few frames of dynamic PET images. For regional brain analysis, PET data was co-registered with the mouse brain T2 MRI template and the modified Mirrione mouse brain atlas^21^ was used to generate VOIs of the following brain regions: cortex, thalamus, cerebellum, basal forebrain septum, hypothalamus, brain stem, central grey matter, olfactory bulb, amygdala, midbrain, corpus callosum, striatum and hippocampus. The data were extracted as time-activity curves and standard uptake values (SUVs) were calculated as concentration in the VOI divided by the injected dose divided by the animal weight.

Five kinetic models were used to estimate the transfer rate constant from arterial plasma to brain tissue (*K_1_*) and *V_T_* in different regions of the brain: the one-tissue compartment (1-TCM), two-tissue compartment (2-TCM), two-tissue compartment with vascular trapping (2-TCM vasc.), spectral analysis and Logan plot (where t* was fixed at 48.92 min for arterial input function measured with ABS, 28.08 min for IDIF vena cava, 33.08 min for IDIF left ventricle and 36.42 min for population average whole blood curve). Each model was fitted using four different input functions: arterial input function (AIF) measured with automated blood sampling system, IDIF from vena cava VOI, IDIF from the left ventricle of the heart and population average whole blood curve. Plasma-to-whole blood ratios were derived (equation (1), supplemental materials) per experimental group with plateau values as follow: Vehicle plasma:whole-blood = 0.11; *Dose 1* and *2* groups plasma:whole-blood = 0.11; *Dose 3* plasma: whole-blood = 0.62 and *Dose 4* plasma:whole-blood = 0.96. In modelling [^18^F]LW223 mouse brain PET data, the impact of *v_B_* on model fits was assessed (data not shown) and it was found it did not improve 1- and 2-tissue model fits. Consequently, the *v_B_* was set to 0.05 across all models. The goodness of fit was assessed by the Akaike Information Criterion (AIC), where the model with lowest AIC and % standard error (%SE) of *K_1_* and *V_T_* was selected as the preferred model. The Lassen plot method was used to estimate *V_ND_*.^22^

In order to determine if the *SUV*s could be reliably used to analyse [^18^F]LW223 brain PET data in healthy mice, correlation analysis was performed between *V_T_* of brain regions measured with 2-TCM (invasive AIF) and average *SUV*s. In addition, *V_T_* apparent (*V_Tapp_*) was calculated by dividing the concentration of [^18^F]LW223 in the brain (90–120 minutes) by the concentration of [^18^F]LW223 measured in plasma at 120 minutes post injection. *SUV* and *V_Tapp_* of peripheral organs (heart, lungs, kidneys, adrenals and spleen) were also calculated.

Percentage target occupancy (%TO), equilibrium dissociation constants (*K_D_*) and lower dose limits *D_5_* and *D_10_* were estimated as described in the supplemental materials.

### Graph plotting and statistical analysis

Plotting of graphs and statistical analysis were performed using Prism 9.3.1 (GraphPad, USA). Normality was investigated using the D’Agostino-Pearson test. One-way ANOVA with Dunnett’s post hoc test, two-way ANOVA or mixed effects model with Šidak’s post hoc test (alpha = 0.05) were used as indicated in the relevant figure legends.

## Results

### [^18^F]LW223 has low radiometabolism in vivo in mice

**Figure 1.**
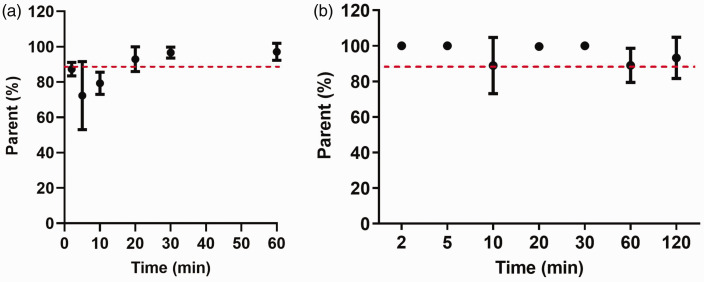
[^18^F]LW223 fraction in mouse plasma (a) and brain tissue (b) samples over time. Data presented as mean ± SD, n = 3 per time point.

Whole blood and plasma activity curves measured using a γ counter (Supplemental Fig. 1 A) showed that the plasma/whole-blood ratios for [^18^F]LW223 were on average 0.75 at 2 minutes and decreased to 0.08 at 120 minutes post-injection (Supplemental Fig. 1B). [^18^F]LW223 metabolism in murine plasma was low up to 60 minutes post injection (Figure 1(a)) and was found to be below the detection limits of the radio-HPLC system at 120 minutes post-injection. Therefore, for the subsequent radiometabolite correction during kinetic modelling, the average parent fraction (measured between 2 and 60 minutes) of 0.83 was used instead of metabolite curve. In the brain, the parent radiotracer was found to be >90% on average (Figure 1(b)). In the heart, lungs, spleen, adrenals and kidneys, the radiotracer was stable up to 120 minutes post-injection (Supplemental Fig. 2 and 3). The highest percentage of radiometabolites was present in the liver, suggesting it may be responsible for the metabolism of [^18^F]LW223 (Supplemental Fig. 2 and 3).

### [^18^F]LW223 has low V_ND_ in the murine brain

**Figure 2.**
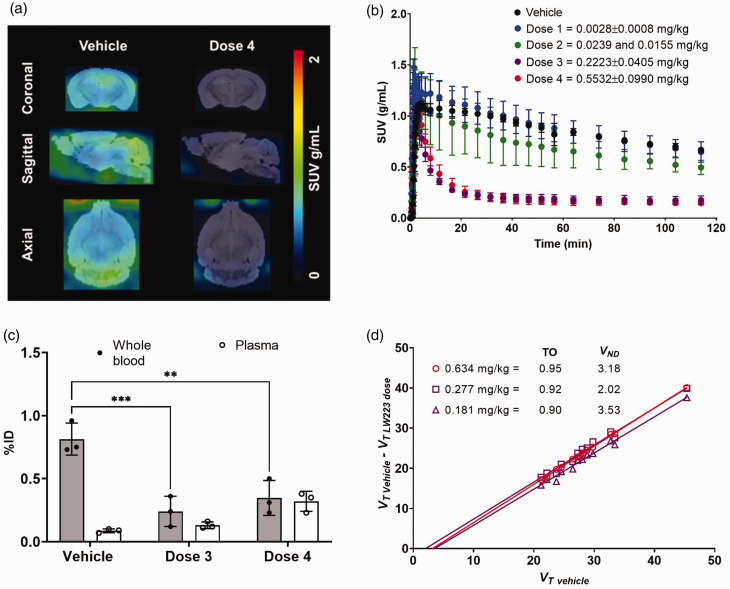
[^18^F]LW223 binding kinetics in murine brain. (a) Representative [^18^F]LW223 *SUV_90–120min_* brain images of a mouse injected with vehicle and the highest dose (i.e. *Dose 4*) of LW223. (b) Time-activity curves of [^18^F]LW223 in the brain of mice co-injected with vehicle (n = 3), *Dose 1* (n = 5), *Dose 2* (n = 2), *Dose 3* (n = 3) and *Dose 4* (n = 3) (mean ± SD) of LW223. (c) Percentage injected dose (%ID) of [^18^F]LW223 in mouse whole blood and plasma at 120 minutes post-injection in vehicle, *Dose 3* and *Dose 4* groups (n = 3/group, mean ± SD; two-way ANOVA, Šidak’s multiple comparison test, alpha = 0.05; **p = 0.0022, ***p = 0.0006) and (d) *V_ND_* and target occupancy (TO, fraction) of [^18^F]LW223 in three individual mouse brains estimated using Lassen plot and 2-TCM. Each point in the Lassen plot represents a different brain region (13 regions in total).

[^18^F]LW223 was able to successfully cross the blood-brain barrier and enter the murine brain (Figure 2(a)). Binding of [^18^F]LW223 to brain was blocked by unlabelled LW223 in a dose-dependent manner (Figure 2(b)). Plasma/whole-blood ratios of [^18^F]LW223 increased when higher doses of LW223 were administered as a result of a significant decrease in binding to cells in the whole-blood component (Figure 2(c)). Plasma/whole-blood ratios in the vehicle, *Dose 3* and *Dose 4* groups were 0.11, 0.54, and 0.92 respectively. Consequently, accurate kinetic modelling required corrections for plasma/whole-blood ratio changes due to changes in radiotracer binding kinetics in the blood pool. Measured plasma/whole-blood ratios were used as plateau values in the one phase exponential decay equation (Supplemental Fig. 1) in order to extrapolate plasma/whole-blood ratios at earlier time points in animals injected with blocking doses of LW223 for kinetic modelling and *V_ND_* estimates.

The *V_T_* and *K_1_* values of [^18^F]LW223 in different brain regions were calculated using different kinetic models (Supplemental Tables 1–2 and Supplemental Figures 4–5), and the 2-TCM with invasive AIF was selected as a preferred kinetic model based on AIC and %SE results. This agrees with previously published data reporting [^18^F]LW223 binding kinetics in the rat brain.^18,23^ Using this model, the average *V_T_* in the whole brain of a mouse injected with vehicle was 29.19 ± 7.76 mL/cm^3^ (mean ± SD, n = 3). In the regional brain analysis, *V_T_* was found to be the highest in the cerebellum, olfactory bulb and brain stem, with the lowest values in the basal forebrain septum, hypothalamus and striatum (Table 1). Data showed that the V*_ND_* of [^18^F]LW223 in the mouse brain was 2.91 ± 0.65 mL/cm^3^ (n = 3, mean ± SD, Figure 2(d)), accounting only 9.97 ± 2.21% of the *V_T_*.

**Table 1.** A summary of *K_1_* and *V_T_* measurements across healthy mouse brain regions when vehicle was co-injected with [^18^F]LW223.

Brain region	*K_1_*	*%SE K_1_*	*V_T_*	*%SE V_T_*
Cortex	2.04 ± 0.9	60.96 ± 71.57	28.04 ± 8.33	9.93 ± 757
Thalamus	2.24 ± 0.84	14.88 ± 3.34	24.54 ± 8.77	3.77 ± 0.4
Cerebellum	2.65 ± 0.69	13.77 ± 7.26	45.37 ± 14.44	7.93 ± 5.32
Basal forebrain septum	3.97 ± 2.88	236.18 ± 310.49	21.2 ± 8.19	5.31 ± 1.52
Hypothalamus	1.84 ± 0.4	10.07 ± 0.63	23.72 ± 8.25	5.42 ± 1.43
Brain stem	2.48 ± 0.35	9.56 ± 2.11	33.43 ± 11.69	3.04 ± 0.59
Central grey matter	2.09 ± 0.49	17.79 ± 5.03	28.02 ± 9.07	7.75 ± 1.72
Olfactory bulb	1.79 ± 0.17	14.63 ± 7.67	35.39 ± 13.53	9.35 ± 2.49
Amygdala	1.42 ± 0.41	7.62 ± 1.99	26.4 ± 8.52	10.23 ± 6.15
Midbrain	2.12 ± 0.14	8.09 ± 1.33	27.27 ± 10.46	4.75 ± 0.56
Corpus callosum	1.47 ± 0.35	12.98 ± 5.85	32.73 ± 10.81	22.79 ± 21.18
Striatum	1.67 ± 0.39	8.68 ± 2.82	22.16 ± 6.64	4.55 ± 2.07
Hippocampus	2.18 ± 0.63	9.76 ± 1.08	28.83 ± 9.3	5.92 ± 1.53

The constants were estimated 2-tissue compartment (2-TC) model with invasively measured arterial input function. Data shown as mean ± SD, n = 3.

### [^18^F]LW223 brain PET data can be quantified using V_Tapp_ or SUV in naïve mice

**Figure 3.**
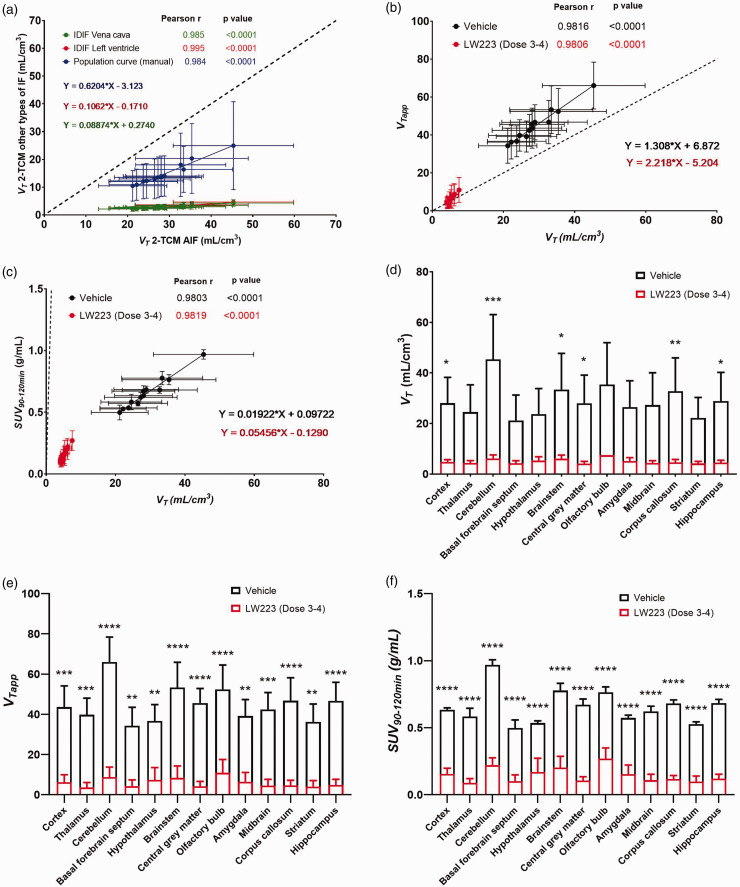
Simplified methods of analysis for quantification of [^18^F]LW223 brain PET data. (a) Comparison of *V_T_* estimated using invasive AIF versus non-invasive IDIF. (b) Correlation graph of *V_T_* versus *V_Tapp_*. (c) Correlation graph of *V_T_* versus *SUV_90–120min_.* Animals co-injected with vehicle (black, n = 3) and Dose 3–4 of LW223 (red, n = 3). Each point represents mean ± SD of *V_T_* (n = 3) in a different brain region (13 regions in total). Pearson *r*, *p* value of each correlation and linear regression equations for each method are indicated in corresponding colours. Dashed black line represents line of identity. Uptake of [^18^F]LW223 across brain regions of mice co-injected with vehicle (n = 3) or *Dose 3–4* of unlabelled LW223 (n = 3), expressed as (d) *V_T_* , (e) *V_Tapp_* and (f) *SUV_90–120min_.* Mean ± SD; for *V_T_* – *p < 0.045, **p = 0.008, ***p = 0.0002, mixed effects model, Šidak’s post hoc test, alpha = 0.05; for *SUV_90–120min_* and *V_Tapp_* – **p < 0.006, ***p < 0.0005, ****p < 0.0001, two-way ANOVA, Šidak’s post hoc test, alpha = 0.05.

Population-based and IDIF (i.e. simplified input functions) resulted in *V_T_* values that positively, linearly and significantly correlated with those derived using individual AIFs (Pearson *p < 0.0001*, Figure 3(a)), albeit with quantitative bias of 38% (population-based) and 90% (IDIF). Furthermore, both *SUV_90–120min_* (determined to be the preferred averaging interval versus invasive methods, Supplemental Fig. 6) and *V_Tapp_* of [^18^F]LW223 significantly correlated with *V_T_* values, with *V_Tapp_* (Figure 3(b)) resulting in overall lower bias when compared to *SUV_90–120min_* (Figure 3(c)). Specifically, *V_Tapp_* resulted in 1.31-fold (vehicle) and 2.22-fold (LW223 *Dose 3–4*) overestimation, and *SUV_90–120min_* in 50-fold (vehicle) and 20-fold (LW223 *Dose 3–4*) underestimation when compared to *V_T_*.

Regional analysis of [^18^F]LW223 distribution in murine brain showed that homologous blocking with LW223 significantly reduced the uptake of [^18^F]LW223 in mouse cortex, cerebellum, brainstem, central grey matter, corpus callosum, and hippocampus, when using any of the outcome measures investigated, i.e. *V_T_* (“gold standard”), *SUV_90–120min_* and *V_Tapp_* (Figure 3(d) to (f)).

### [^18^F]LW223 has a high affinity and upper mass dose limits in various murine tissues in vivo

**Figure 4.**
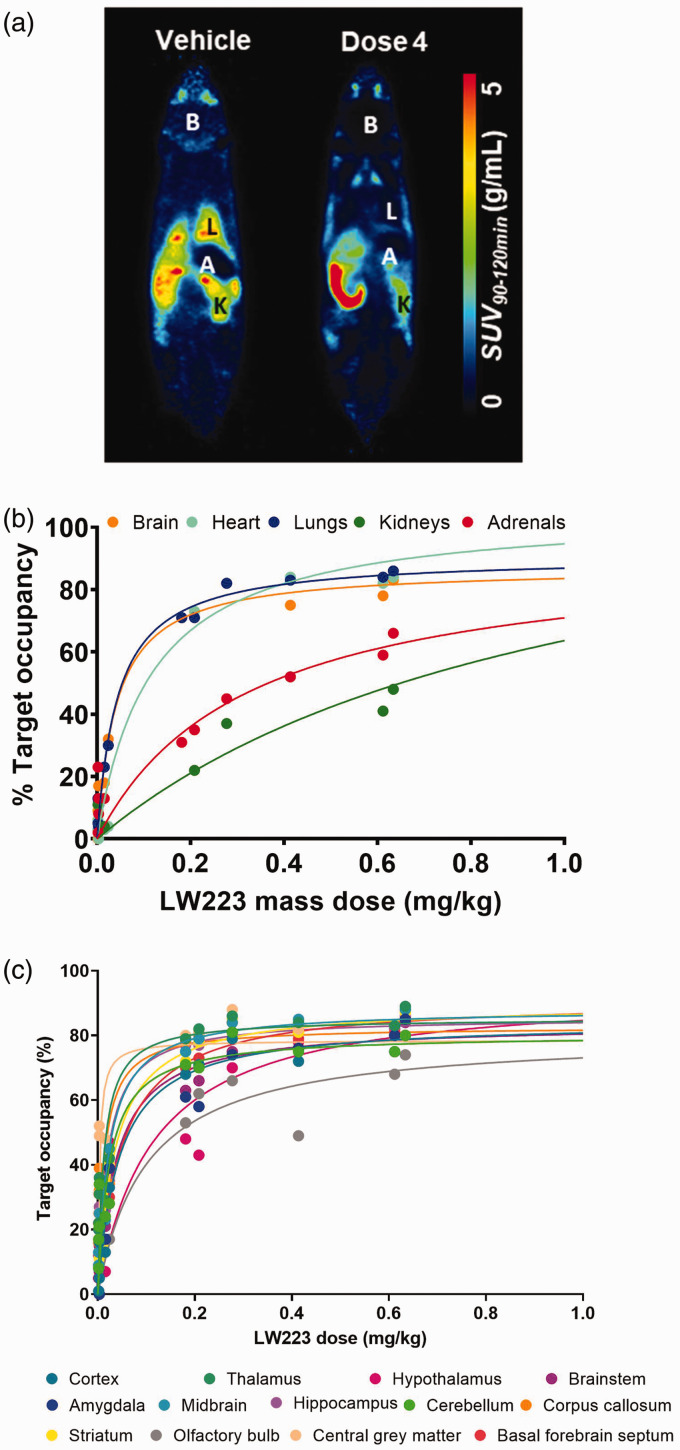
Whole-body binding kinetics of [^18^F]LW223 in mice. (a) Representative [^18^F]LW223 average SUV at 90–120 min total-body images of vehicle (left) and LW223 *Dose 4* (right) injected mice. Organ of interest annotated as follows: B–brain, L–lungs, K–kidneys, A–adrenals. (b) LW223 saturation plots in mouse brain, heart, lungs, kidneys and adrenals. (c) LW223 saturation plots for mouse brain regions. Target occupancy was calculated as described in the methods section and on the supplementary methods file. All saturation plots fitted using one site specific binding equation. Each point represents a different mouse individual dose (n = 10 to 13 different doses) with %occupancy values <0% excluded from fit.

Binding of [^18^F]LW223 to various TSPO rich tissues throughout the mouse body was reduced following homologous blocking with LW223 (Figure 4(a)) and this reduction was dose dependent at whole organ level (Figure 4(b) and Supplemental Fig. 7) as well as individual brain regions (Figure 4(c)). Estimated *K_d_* values in different brain sub-regions (Supplemental Table 3) and various organs throughout the body (Table 2) ranged between 0.03 (brain) and 1.02 mg/kg (kidney). Fitting of the dose-response curves allowed for calculation of LW223 *D_5_* and *D_10_* (Table 2), which are important parameters for setting radiotracer specifications for preclinical and clinical use.

**Table 2.** Estimated equilibrium dissociation constant (*K_d_*), lower mass dose limits *D_5_* and *D_10_* of LW223 across multiple organs and brain regions of healthy adult male C57Bl/6J mice.

Tissue	*K_d_* (mg/kg)	*D_5_* (mg/kg)	*D_10_* (mg/kg)
Brain	0.04	0.0024	0.0051
Heart	0.12	0.0055	0.0117
Lungs	0.04	0.0023	0.0048
Kidneys	1.02	0.0501	0.1038
Adrenals	0.32	0.0226	0.0478

## Discussion

The main aims of this study were to quantify the *V_ND_* of [^18^F]LW223 in the murine brain and assess the potential for using simplified strategies for quantification of brain TSPO using [^18^F]LW223 and PET imaging. The results presented herein demonstrate that [^18^F]LW223 had the lowest *V_ND_* reported thus far for a TPSO PET radiotracer using a homologous blocking study design. Furthermore, we found that [^18^F]LW223 had high target occupancy and affinity for TSPO in different organs throughout the whole-body of healthy mice. *D_5_* and *D_10_* calculated from collected data confirmed the molar activity of [^18^F]LW223 is excellent. This shows that [^18^F]LW223 respects the radiotracer principle, which is acutely important when imaging small animals like mice, and augments translational potential of this novel TSPO PET radiotracer. Importantly, data reported here showed that simplified outcome measures, namely SUV and *V_Tapp_*, can be successfully deployed for quantification of [^18^F]LW223 PET data in different organs throughout the mouse body. This is enabled by the low *V_ND_* of this radiotracer coupled with low radiometabolism in blood and tissue.

Radiometabolite analysis showed that [^18^F]LW223 had a favourable metabolic profile, with >90% of parent compound measured in TSPO-expressing organs up to two hours and >80% in plasma up to one hour post-intravenous injection. This is among the highest values reported for TSPO PET radiotracers, with [^18^F]DPA-714 showing 44% parent in plasma (1 hour p.i.) and 72% parent in brain (2 hours p.i) (mice);^24^ [^18^F]FEPPA – 35% parent in plasma (1 hour p.i.) and 77% parent in brain (2 hours p.i.) (mice);^25^ (*S*)-[^18^F]GE387 – 28% parent in plasma (1 hour p.i.) and 78% parent in brain (1 hour p.i.) (mice);^26^ [^11^C]PBR28 – 50% parent in plasma (5 minutes p.i.) (rats);^27^ *(R)-*[^11^C]PK11195 – 50% parent in plasma (21 minutes p.i.) (rats).^27^ Data also showed that [^18^F]LW223 is predominately metabolised in the liver, since the highest proportion of radiometabolites was measured in this organ (>60% parent). This finding was in line with metabolic studies performed using another TSPO PET radiotracer, [^18^F]DPA-714, showing that it is also metabolised in the liver.^24^

The intracellular localisation of TSPO requires lipophilic compounds that are prone to accumulation in the non-specific and non-displaceable compartments of a tissue, which often results in high *V_ND_*. This prevents early and accurate detection of changes in TSPO expression, making current TSPO PET radiotracers more suitable to image advanced pathology.^28^ Our results showed that the *V_ND_* of [^18^F]LW223 in a mouse brain was low, contributing only to ∼10% of *V_T_*. Other currently available TSPO PET radiotracers have higher percentage *V_ND_* values, although these are based on human data using heterologous blocking design ^14,16,29,30^ and polymorphism plots.^15^ Furthermore, a head-to-head comparison to other TSPO radiotracers using heterologous blocking in the same species and experimental conditions is needed to directly compare [^18^F]LW223 *V_ND_* results with those obtained with currently available TSPO radiotracers. *V_ND_* estimation is not common in preclinical small animal PET due to challenges in obtaining arterial input functions in mice and measuring individual *f_p_* values due to the mouse limited circulating blood volume. Notwithstanding, in humans, the *V_ND_* for [^11^C]ER176 is 20%,^29^ [^18^F]DPA714 is ∼45%,^30^ [^11^C]PBR28 is ∼30%,^14^ [^11^C]-(*R*)-PK11195 is 57% ^15^ and [^18^F]GE-180 is 55%.^16^ Despite the potential for species differences and quantification strategies used to derive *V_ND_*, [^18^F]LW223 data presented here indicates that this new radiotracer is in a unique position with regards to suitable properties for *in vivo* brain imaging. Importantly, recent data have also shown that *V_ND_* of [^11^C]PBR28 in the brain can differ between patient groups and conditions and should, therefore, always be considered were possible.^19^ Overall, the low *V_ND_* of [^18^F]LW223 in the brain may reduce confounding effects due to high radiotracer non-specific/non-displaceable binding when quantifying disease activity in different pathophysiological contexts and enable high sensitivity imaging for early detection of brain disease. Results reported here provide key proof-of-concept evidence of [^18^F]LW223 excellent performance in the murine brain and future studies can now follow to explore sex-differences with this new and promising TSPO radiotracer.

Based on the findings reported herein, [^18^F]LW223 PET data collected between 90–120 minutes post-injection and expressed as *SUV_90–120min_* or corrected for terminal plasma radioactivity and expressed as *V_Tapp_* provided robust quantitative measures of TSPO expression in a healthy murine brain, with *V_Tapp_* having lower bias than *SUV_90–120min._* The use of *SUV_90–120min_* or *V_Tapp_* resulted in reduced inter-subject variability when compared to *V_T_*, which might be due to challenges associated with generating robust individual arterial input functions in small animals,^31^ albeit with an expected quantitative bias.^23,32,33^ The use of simplified outcome measures also provide opportunity to robustly quantify whole-body TSPO PET data. This widens the scope for applications of [^18^F]LW223 and opens new avenues to study biological TSPO axes, such as the heart-brain axis.^18,34^ Notwithstanding, the use of these simplified [^18^F]LW223 PET outcome measures also needs to be validated in animal models of inflammation, as they might not be compatible with changes in blood TSPO expression^35,36^ and/or blood-brain barrier permeability^37^ induced by harmful stimuli. Therefore, the use of [^18^F]LW223 simplified outcome measures, or in fact any TSPO PET radiotracer, should be validated in a case by case scenario for each pathology of interest.

In conclusion, the results of this study demonstrated that [^18^F]LW223 has the physicochemical properties of a successful PET radiotracer, including low *V_ND_*, with high affinity for target throughout the body. Additionally, the simplified outcome measures validated in this study will enable wider adoption of this imaging approach in the future.

## Supplemental Material

sj-pdf-1-jcb-10.1177_0271678X231205661 - Supplemental material for [^18^F]LW223 has low non-displaceable binding in murine brain, enabling high sensitivity TSPO PET imaging Click here for additional data file. Supplemental material, sj-pdf-1-jcb-10.1177_0271678X231205661 for [^18^F]LW223 has low non-displaceable binding in murine brain, enabling high sensitivity TSPO PET imaging by Agne Knyzeliene, Mark G MacAskill, Carlos J Alcaide-Corral, Timaeus EF Morgan, Martyn C Henry, Christophe Lucatelli, Sally L Pimlott, Andrew Sutherland and Adriana AS Tavares in Journal of Cerebral Blood Flow & Metabolism
