# Supplementary material for: [18F]LW223 has low non-displaceable binding in murine brain, enabling high sensitivity TSPO PET imaging
Source: J Cereb Blood Flow Metab. 2023 Oct 5;44(3):397–406. doi: 10.1177/0271678X231205661 (PMC10870961; doi:10.1177/0271678X231205661)
Supplement: sj-pdf-1-jcb-10.1177_0271678X231205661 - Supplemental material for [18F]LW223 has low non-displaceable binding in murine brain, enabling high sensitivity TSPO PET imaging [file sj-pdf-1-jcb-10.1177_0271678X231205661.pdf]

$[^{18}F]$LW223 has low non-displaceable binding in murine brain, enabling high sensitivity TSPO PET imaging

Agne Knyzeliene[1,2], Mark G. MacAskill[1,2], Carlos J. Alcaide-Corral[1,2], Timaeus E. F. Morgan[1,2], Martyn C. Henry[3], Christophe Lucatelli[2], Sally L. Pimlott[4], Andrew Sutherland[3], Adriana A. S. Tavares[1,2]*

Affiliations:

[1]BHF-University of Edinburgh Centre for Cardiovascular Science, University of Edinburgh, UK

[2]Edinburgh Imaging, University of Edinburgh, UK

[3]School of Chemistry, University of Glasgow, UK

[4]West of Scotland PET Centre, Greater Glasgow and Clyde NHS Trust, UK

*Corresponding author. Email: adriana.tavares@ed.ac.uk (Adriana A.S. Tavares)

SUPPLEMENTAL MATERIALS

Surgical cannulation of the femoral vein and artery:

Healthy adult male mice were anaesthetised using 2% isofluorane gas (Isoflo® APIECE, Zoetis, UK) in a 1L/min 50/50 oxygen/nitrous oxide mixture. Then, using a stereomicroscope (Carl Zeiss, Germany), PE10 catheters (Fisher Scientific, UK) filled with 20 IU heparinised saline (heparine: B. Braun Medical Inc., UK; saline: Hameln Pharmaceuticals, UK) were surgically inserted into femoral artery and vein for automated blood sampling (ABS) and administration of a radiotracer, respectively. The catheters were fastened with ligatures (6-0 silk thread) and secured in place using surgical glue. During the surgery, the body temperature was maintained using a heated mat. Arterial and venous catheters were connected to the automated blood sampling (ABS) system (Twilite, SwissTrace, Switzerland) for invasive input function measurements. For radiometabolite experiments, only the femoral artery was cannulated following the same procedure described above.

Radiometabolite studies in mice:

The radioactivity in whole blood samples was measured using a gamma (γ) counter (2470 Wizard2™, Perkin Elmer, UK). The heart, lung, brain, spleen, liver, kidney and adrenal tissue samples were homogenised using IKA T10 Basic Ultra-Turrax homogeniser with 5 mm diameter dispersing tool (IKA® England LTD, UK). In order to facilitate the process, 1 mL of ultrapure deionised H_2O (Barnstead Smart2Pure, Thermo Fisher Scientific, UK) was added to each sample tube prior to homogenisation. The activity in 200 µL of each tissue homogenate was measured using a γ counter. Whole blood samples (500 µL) were centrifuged for 5 minutes at 2,000 × g and 4 °C to separate plasma. The activity in the resulting plasma sample aliquots was measured on a γ counter.

Whole blood and plasma data obtained using a γ-counter was converted from dpm/mL to kBq/mL and normalised for injected dose to generate a population average input function. The data was also used to generate plasma-to-whole blood ratios at different time points post-[^{18}F]LW223 injection which was then fitted using one phase decay model:

$$Y=(Y_0\text{-Plateau})\cdot e^{(-K\times X)} + \text{Plateau} \qquad \text{Eq.1}$$

Using Eq. 1 above, plasma-to-whole blood ratios were extrapolated for all *in vivo* PET imaging time points and used for correction of whole blood data during kinetic modelling to generate plasma input functions.

The plasma samples were denatured by adding 100% acetonitrile at 1:1.4 (v:v) ratio, followed by brief mixing using a vortex and centrifugation for 4 minutes at 2,000 × g and 4 °C. The activity in the supernatant as well as cell pellets was measured using a γ counter. The rest of the supernatant was collected for the radio-high performance liquid chromatography (HPLC) analysis. The homogenised

tissue samples were denatured adding 100% acetonitrile at 1:1.4 (v:v) ratio, followed by brief stirring using vortex and centrifugation for 4 minutes at 2,000 × g and 4 °C. The activity in the 200 µL of supernatant as well as cell pellets was measured using a γ counter. The rest of the supernatant was collected for the HPLC analysis.

The supernatants of blood and tissue samples were analysed using UHPLC system (Dionex Ultimate 3000, ThermoFisher, UK). The chromatographic separation was carried out at 25 °C using a Luna® C18(2) 250 mm × 10 mm, 10 µm column (Phenomenex, UK). The mobile phase consisted of 70% acetonitrile in ultrapure deionised water. Processed blood and tissue samples were injected onto the HPLC (injection volume of 100 µL) and the column was eluted with an isocratic method and 4 mL/min for 15 minutes. [^{18}F]LW223 retention time and peak area in the radiotracer sample (used for confirmation of parent compound retention time) and in radioactive tissue/blood samples were detected by a Flow Star LB514 gamma detector (Berthold Technologies, UK). The chromatograms were manually integrated for quantification of the peak area using Chromeleon version 7 (ThermoFisher, UK). The percentage parent fraction in plasma and tissue samples at each time point post-[^{18}F]LW223 injection was used as the outcome measure.

Target occupancy (TO), K_d, D_5 and D_{10} estimates:

Percentage target occupancy (%TO) was determined by the following formula:

$$\%TO = \frac{SUV_{90\text{-}120min}(vehicle) - SUV_{90\text{-}120min}(Dose\ X)}{SUV_{90\text{-}120min}(vehicle)} \times 100 \qquad \text{Eq. 2}$$

where *Dose X* stands for either *Dose 1, 2, 3* or *4*. $SUV_{90\text{-}120\ min}$ was used for %TO estimation as plasma activity measurements were not collected for all the animals and this was the only tested outcome measure which did not require plasma data for its estimation. The %TO values for brain (global and regional), heart, lungs, kidneys, adrenals and spleen were fitted using one-site specific binding model with extrapolation of the data to 1 mg/kg LW223 to generate saturation curves and estimate equilibrium dissociation constant K_D:

$$Y = \frac{B_{max} \times X}{K_D + X} \qquad \text{Eq. 3}$$

where X is radioligand concentration, Y is %TO and B_{max} is the maximum %TO.

The negative %TO values that exceeded variability of vehicle data for that organ/brain region were excluded when plotting saturation curves. Lower dose limits D_5 and D_{10} were also derived by modifying one-site binding into:

$$X = \frac{\left(\frac{Y}{B_{max}}\right) \times K_D}{1 - \left(\frac{Y}{B_{max}}\right)} \qquad \text{Eq. 4}$$

where $Y = 5$ for D_5 or $Y = 10$ for D_{10}, X is the mass dose limit, B_{max} in the maximum %TO and K_D is equilibrium dissociation constant.

SUPPLEMENTAL DATA:

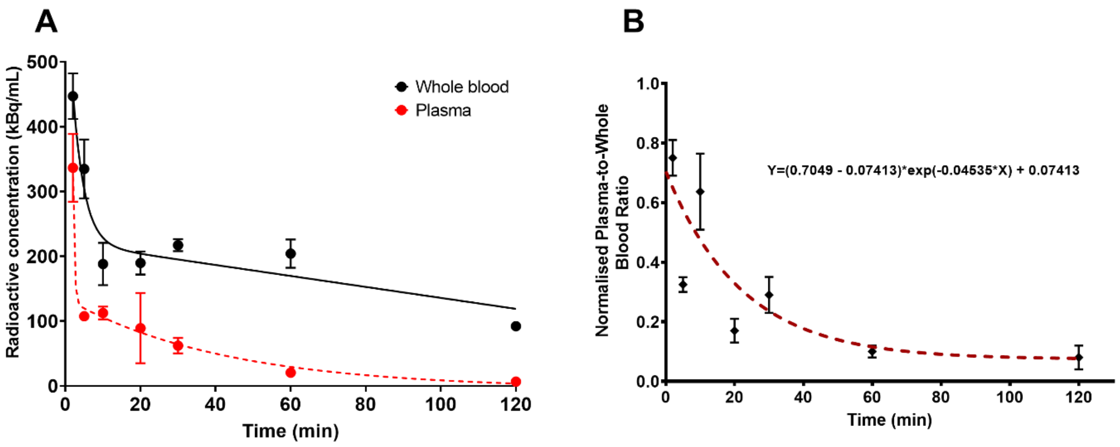

Supplemental Figure 1. [^{18}F]LW223 kinetics in mouse whole blood and plasma. (A) The radioactive concentration of [^{18}F]LW223 in mouse whole blood and plasma over time. Data presented as mean±SD and fitted with two phase exponential decay model. n=2 for 2, 5 and 60 min; or n=3 for 10, 20, 30 and 120 min. **(B)** Plasma to-whole blood ratio of [^{18}F]LW223 over time. Estimation of the ratio at any given time point between 2 and 120 minutes post-[^{18}F]LW223 injection can be done using equation displayed on graph. Data presented as mean±SD and fitted using one phase exponential decay. n=2 for 2, 5, 20, 60, 120 min and n=3 for 60 and 120 min time points.

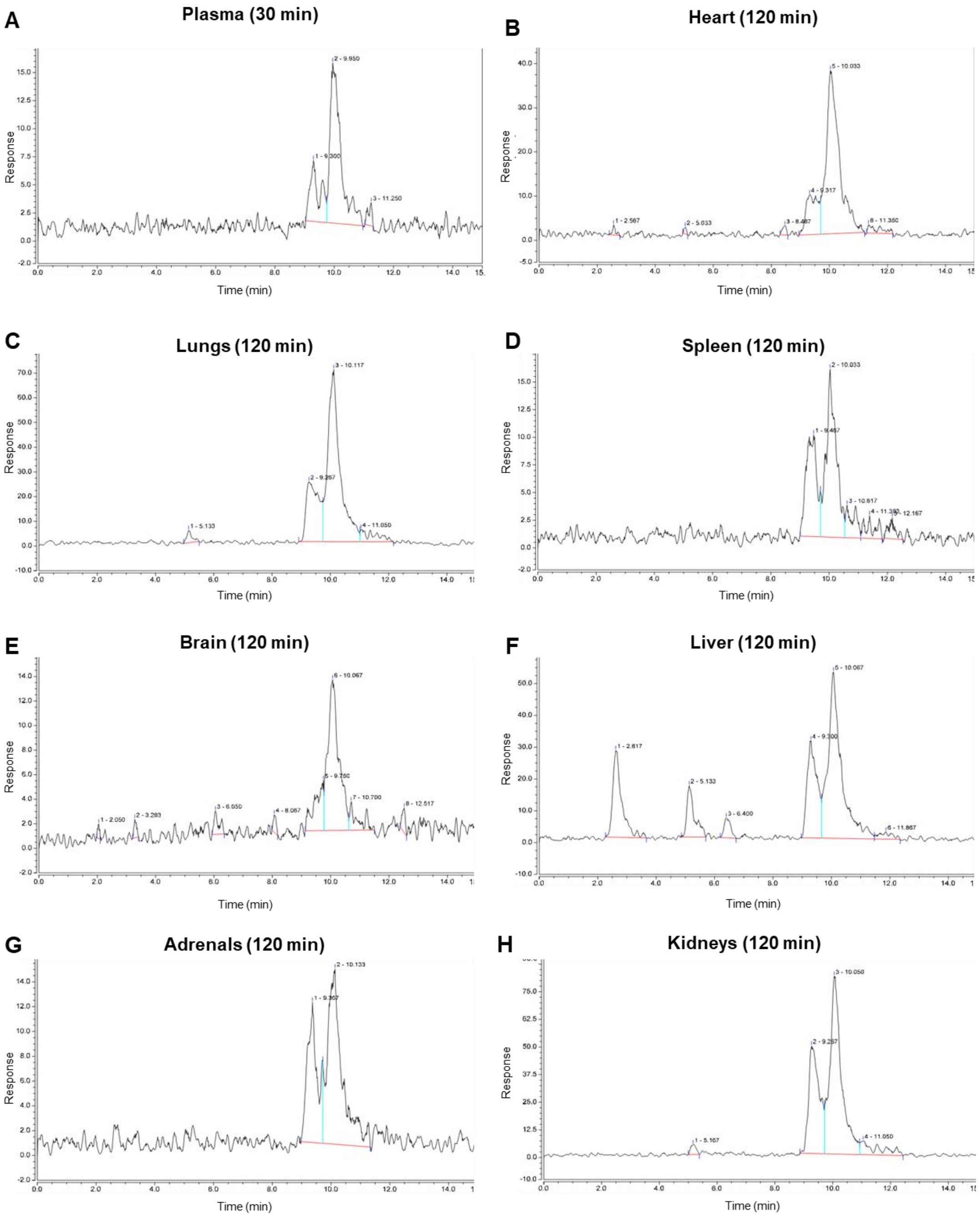

Supplemental Figure 2. Representative chromatograms of [^{18}F]LW223 fraction (retention time between 9 and 10 minutes) and its radiometabolites in mouse **(A)** plasma (30 minutes post-injection), **(B)** heart, **(C)** lungs, **(D)** spleen, **(E)** brain, **(F)** liver, **(G)** adrenals and **(H)** kidneys (B-H 120 minutes post-injection).

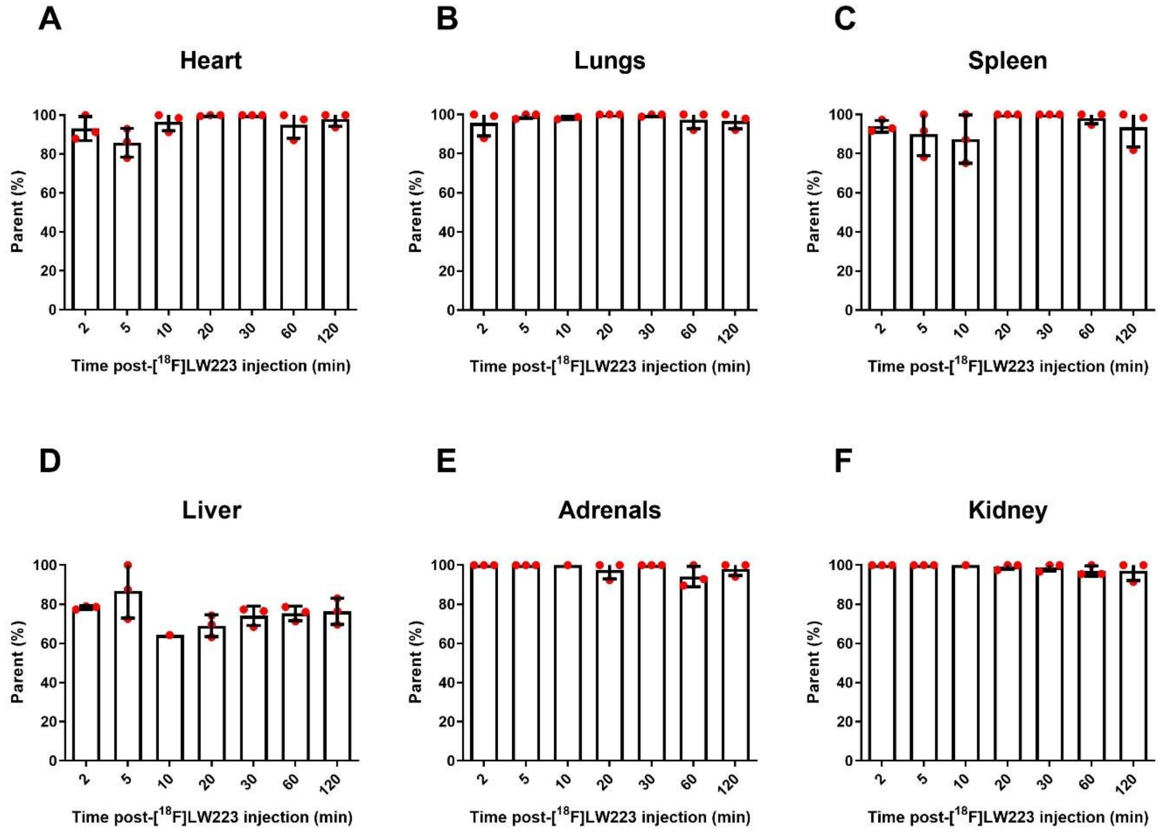

Supplemental Figure 3. *In vivo* [^{18}F]LW223 fraction in C57Bl/6J mouse **(A)** heart, **(B)** lungs, **(C)** spleen, **(D)** liver, **(E)** adrenals, and **(F)** kidneys (n=3, mean±SD; except liver, adrenals and kidneys at 10 minutes post-injection n=1).

Supplemental Table 1. A summary of K_1 and V_T measurements across healthy mouse brain regions (vehicle group) following intravenous bolus injection of [^{18}F]LW223. The constants were estimated using 1-tissue compartment (1-TC), 2-tissue compartment (2-TC), 2-tissue compartment with vascular trapping (2-TC vasc. trapping), Logan plot and spectral analysis kinetic models with invasively measured arterial input function. Data shown as mean±SD, n=3.

Region	1-TCM				2-TCM				2-TCM vasc. trapping				Logan plot		Spectral	
	K_1	%SE K_1	V_T	%SE V_T	K_1	%SE K_1	V_T	%SE V_T	K_1	%SE K_1	V_T	%SE V_T	V_T	%SE V_T	K_1	V_T
**Cortex**	0.55 ±0.14	11.27 ±1.59	18.74 ±6.43	8.71 ±0.89	2.04 ±0.9	60.96 ±71.57	28.04 ±8.33	9.93 ±757	2.08 ±0.75	72.67 ±64.24	14.22 ±8.52	32.81 ±16.5	23.07 ±8.13	11.75 ±12.85	2.46 ±0.68	104.78 ±65.96
**Thalamus**	0.77 ±0.25	10.96 ±1.04	18.8 ±6.77	7.38 ±0.44	2.24 ±0.84	14.88 ±3.34	24.54 ±8.77	3.77 ±0.4	2.4 ±0.95	21.16 ±7.7	18.01 ±6.38	31.04 ±18.68	24.08 ±9.22	3 ±0.66	2.44 ±1.03	46.78 ±15.62
**Cerebellum**	0.76 ±0.24	11.98 ±1.51	28.75 ±10.9	9.67 ±0.78	2.65 ±0.69	13.77 ±7.26	45.37 ±14.44	7.93 ±5.32	2.9 ±0.63	26.26 ±11.6	16.87 ±9.19	20.9 ±7.49	41.71 ±13.28	8.14 ±8.41	3.2 ±0.79	113.25 ±35.76
**Basal forebrain septum**	0.59 ±0.23	10.6 ±1.04	16.28 ±6.07	7.44 ±0.49	3.97 ±2.88	236.18 ±310.49	21.2 ±8.19	5.31 ±1.52	4.04 ±2.86	538.23 ±731.91	14.93 ±6.56	36.01 ±25.11	20.55 ±8.06	3.56 ±1.13	2.07 ±0.6	59.18 ±60.06
**Hypothalamus**	0.77 ±0.14	11.74 ±1.29	16.69 ±6.3	7.81 ±0.13	1.84 ±0.4	10.07 ±0.63	23.72 ±8.25	5.42 ±1.43	1.94 ±0.53	14.02 ±1.72	13.51 ±2.82	31.44 ±13.91	22.85 ±7.94	4.39 ±1.1	1.99 ±0.54	85.88 ±71.39
**Brain stem**	0.79 ±0.2	11.33 ±1.34	23.85 ±9.29	8.21 ±0.59	2.48 ±0.35	9.56 ±2.11	33.43 ±11.69	3.04 ±0.59	2.76 ±0.44	14.55 ±3.91	17.18 ±6.25	13.05 ±2.09	32.49 ±11.84	3.01 ±1.19	2.84 ±0.42	169.23 ±55.79
**Central grey matter**	0.89 ±0.2	11.39 ±1.13	20.77 ±7.25	7.5 ±0.27	2.09 ±0.49	17.79 ±5.03	28.02 ±9.07	7.75 ±1.72	2.18 ±0.58	25.47 ±6.68	17.32 ±3.63	57 ±37.1	27.82 ±10.39	4.71 ±1.07	2.24 ±0.6	129.9 ±66.21
**Olfactory bulb**	0.64 ±0.16	11.68 ±1.27	22.06 ±7.58	9.17 ±0.44	1.79 ±0.17	14.63 ±7.67	35.39 ±13.53	9.35 ±2.49	1.97 ±0.25	30.33 ±15.28	10.82 ±1.66	22 ±8.69	32.32 ±13.18	10.02 ±6.66	2.08 ±0.19	65.86 ±28.86
**Amygdala**	0.67 ±0.17	11.44 ±1.18	16.82 ±6.37	8.05 ±0.31	1.42 ±0.41	7.62 ±1.99	26.4 ±8.52	10.23 ±6.15	1.42 ±0.45	11.03 ±2.46	16.17 ±1.16	147.05 ±93.5	24.02 ±8.66	4.16± 1.12	1.46 ±0.48	68.7 ±40
**Midbrain**	1.01 ±0.07	11.18 ±1.46	19.56 ±8.15	7.65 ±0.68	2.12 ±0.14	8.09 ±1.33	27.27 ±10.46	4.75 ±0.56	2.15 ±0.13	10.31 ±1.75	17.46 ±6.84	27.56 ±3.6	26.15 ±9.85	4.13 ±0.77	2.21 ±0.08	105.79 ±39.97
**Corpus callosum**	0.68 ±0.11	10.76 ±2.89	20.13 ±8.11	7.56 ±1.42	1.47 ±0.35	12.98 ±5.85	32.73 ±10.81	22.79 ±21.18	1.64 ±0.5	36.82 ±9.17	19.28 ±15.61	88.03 ±103.53	24.81 ±9.42	12.83 ±12.39	1.67 ±0.5	116.68 ±68
**Striatum**	0.76 ±0.08	10.51 ±1.74	16.44 ±6.64	7.27 ±1.14	1.67 ±0.39	8.68 ±2.82	22.16 ±6.64	4.55 ±2.07	1.72 ±0.45	11.53 ±4.92	18.12 ±7.59	42.87 ±26.02	21.4 ±6.8	2.24 ±0.75	1.77 ±0.51	25.9 ±3.9
**Hippocampus**	0.87 ±0.18	11.77 ±0.86	20.14 ±7.92	8.4 ±0.47	2.18 ±0.63	9.76 ±1.08	28.83 ±9.3	5.92 ±1.53	2.31 ±0.69	13.92 ±2.95	17.72 ±5.11	56.45 ±54.4	28.52 ±9.89	4 ±3.69	2.37 ±0.74	48.49 ±25.78

Supplemental Table 2. A summary of Akaike information criterion (AIC) across healthy mouse brain regions (vehicle group) calculated following kinetic modelling of [^{18}F]LW223 PET data. The AIC values are shown for 1-tissue compartment (1-TC), 2-tissue compartment (2-TC), 2-tissue compartment with vascular trapping (2-TC vasc. trapping), and spectral analysis kinetic models with arterial input function (AIF). Data shown as mean±SD, n=3.

Region	1-TCM	2-TCM	2-TCM vasc. trapping
Cortex	79.03±3.96	25.36±11.79	26.86±10.7
Thalamus	72.04±5.75	15.81±3.63	17.25±4.63
Cerebellum	81.38±4.2	12.68±12.30	12.45±11.8
Basal forebrain septum	70.64±7	25±9.85	25.23±8.93
Basal forebrain septum	70.64±7	25±9.85	25.23±8.93
Hypothalamus	78.08±5.39	20.29±10.87	31.13±12.64
Brain stem	73.66±8.76	-4.75±9.25	-9.4±11.23
Central grey matter	73.34±3.10	36.17±5.83	38.22±7.72
Olfactory bulb	86.37±6.73	30.15±7.09	30.29±7.36
Amygdala	79.56±5.66	21.52±19.1	23.15±21.66
Midbrain	81.55±12.89	20.6±12.25	22.58±11.82
Corpus callosum	72.88±13.46	33.8±13.56	34.5±12.81
Striatum	79.20±18.21	15.85±17.08	18.61±16.77
Hippocampus	87.23±7	26.43±8.17	27.09±9.28

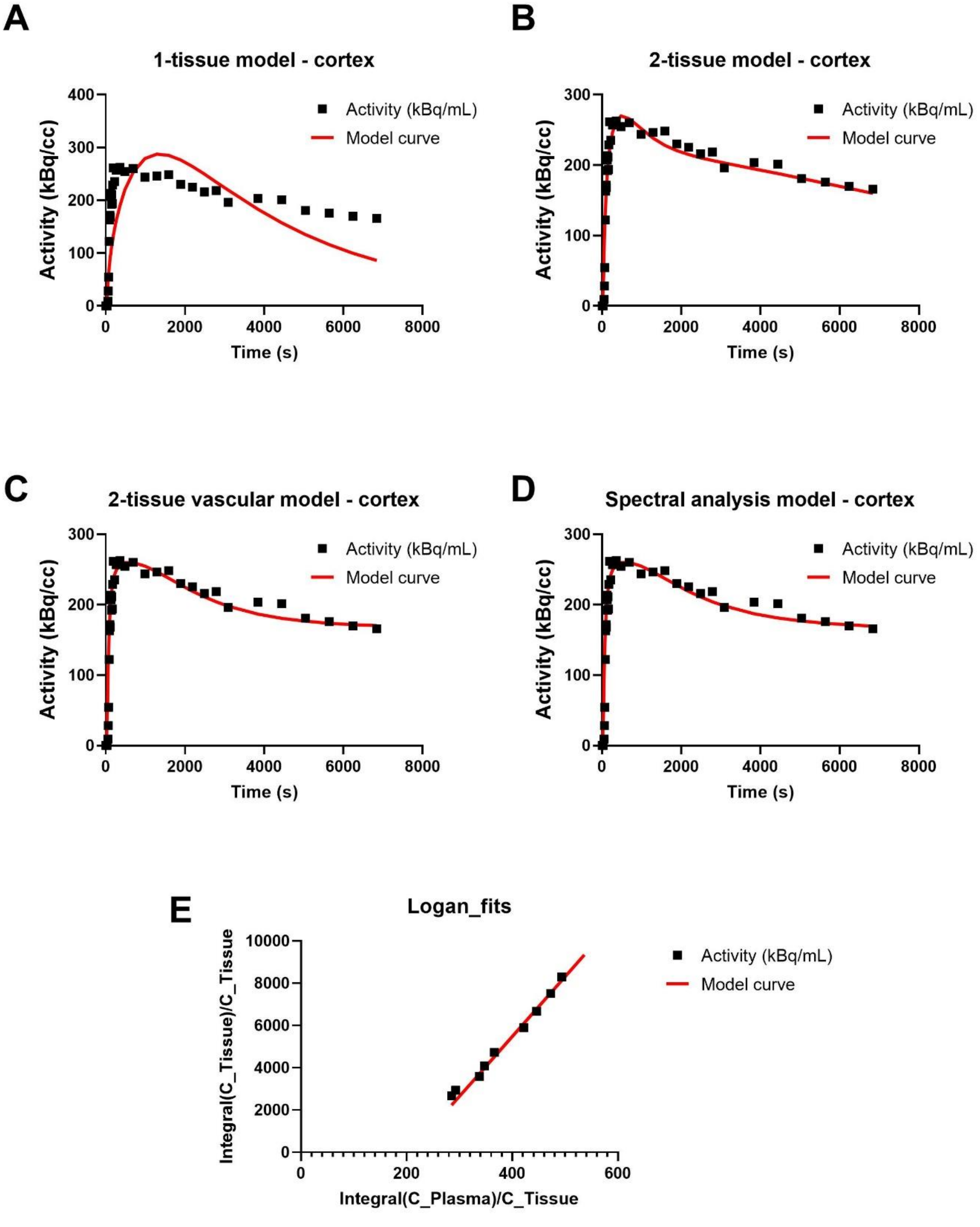

Supplementary Figure 4. Representative examples of different models versus measured data on naïve mouse cortex. **(A)** 1-tissue compartmental modelling. **(B)** 2-tissue compartmental modelling. **(C)** 2-tissue compartmental modelling with vascular trapping. **(D)** Spectral analysis. **(E)** Logan plot with *t**=48.92min.

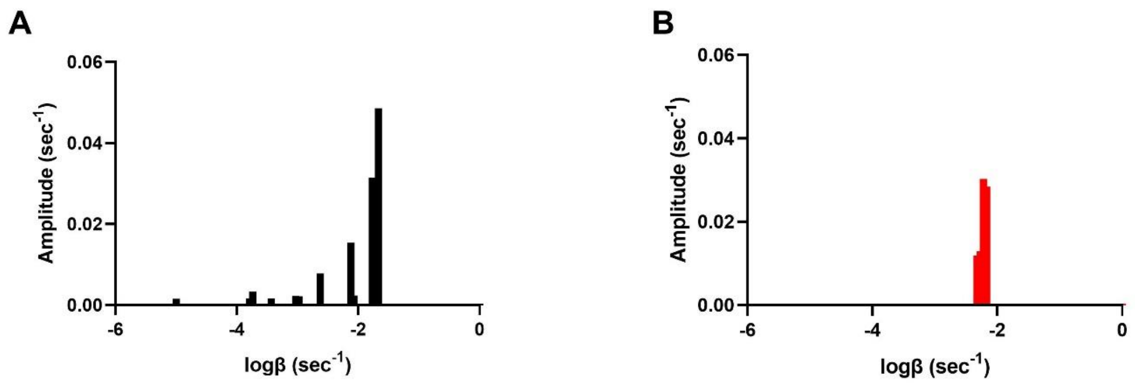

Supplementary Figure 5. Average histogram for spectral analysis of murine cortex for three vehicle control animals **(A)** and three mice administered with saturating blocking concentrations of LW223 **(B)**.

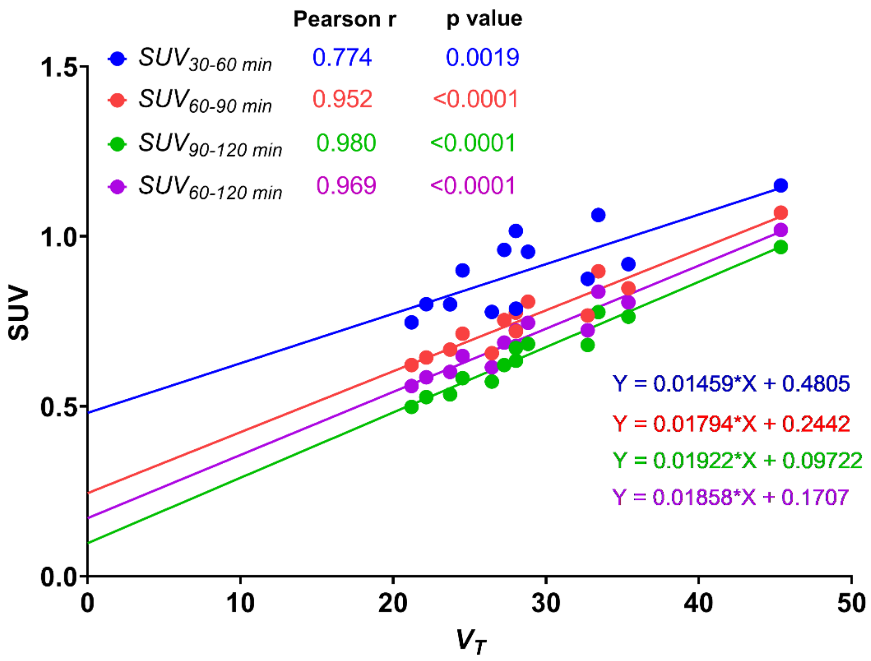

Supplemental Figure 6. Correlation graph between brain V_T values and SUVs averaged at four different imaging periods of the vehicle group. Each plotted data point is a mean of n=3. Each data point represents a different brain region (13 regions in total). Linear equations for each correlation are displayed in corresponding colours.

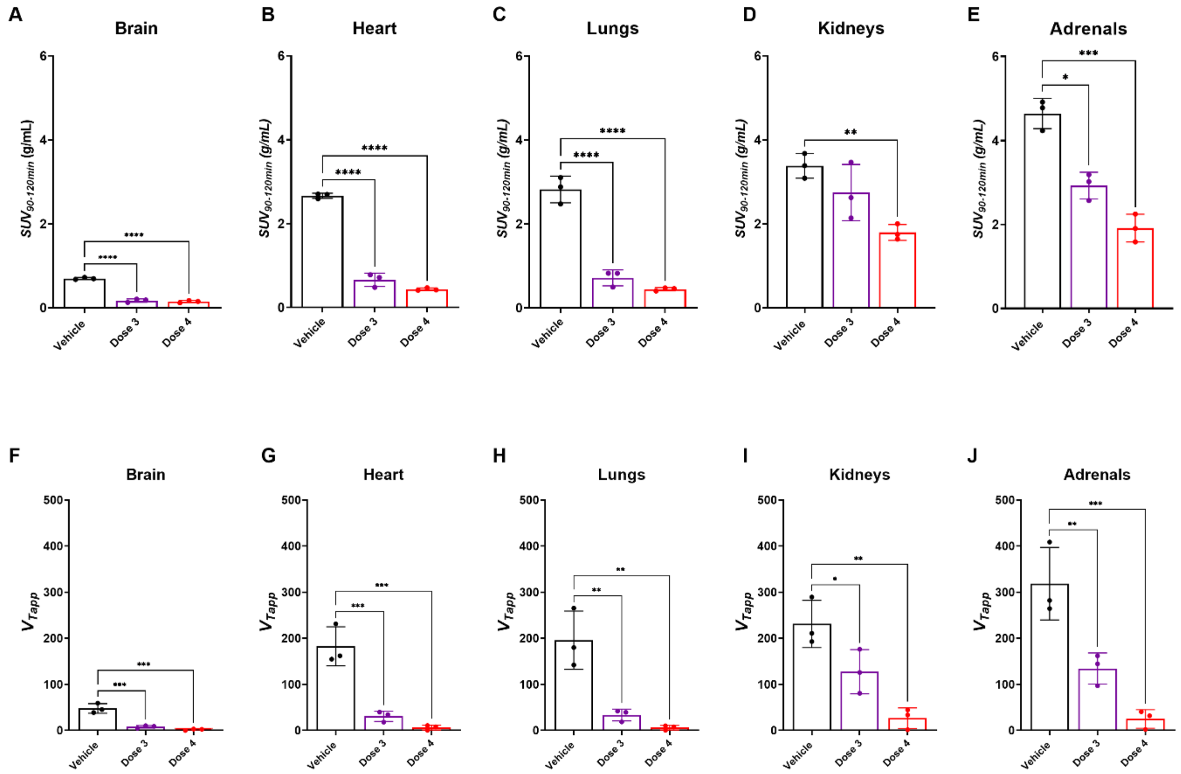

Supplemental Figure 7. The change in $SUV_{90\text{-}120min}$ across LW223 doses (vehicle n=3, dose 3 n=3, dose 4 n=4; mean ± SD) for **(A)** brain, **(B)** heart, **(C)** lungs, **(D)** kidneys and **(E)** adrenals were plotted (one way ANOVA, Dunnett's post hoc test, alpha = 0.05; * p=0.0159, ** p=0.0059, *** p=0.0006, **** p<0.0001). The change in V_{Tapp} across LW223 doses (vehicle n=3, dose 3 n=3, dose 4 n=4; mean ± SD) for **(F)** brain, **(G)** heart, **(H)** lungs, **(I)** kidneys and **(J)** adrenals were plotted (one way ANOVA, Dunnett's post hoc test, alpha = 0.05; * p=0.043, ** p<0.0079, *** p<0.0007).

Supplemental Table 3. Estimated equilibrium dissociation constant (K_d) of LW223 across multiple brain regions of healthy adult male C57Bl/6J mice.

**Tissue**	***Calculated*** $\boldsymbol{K_d}$ **(mg/kg)**
Cortex	0.06
Thalamus	0.04
Cerebellum	0.06
Basal forebrain septum	0.06
Hypothalamus	0.19
Brain stem	0.09
Central grey matter	0.03
Olfactory bulb	0.10
Amygdala	0.11
Midbrain	0.05
Corpus callosum	0.03
Striatum	0.05
Hippocampus	0.04
